# Administration of Recombinant TAPBPL Protein Ameliorates Collagen-Induced Arthritis in Mice

**DOI:** 10.3390/ijms241813772

**Published:** 2023-09-07

**Authors:** Zhenzhen Zhang, Jin Zhao, Kuan Chen Lai, Laijun Lai

**Affiliations:** 1 First Affiliated Hospital of Fujian Medical University, Fuzhou 350004, China; 2 Department of Allied Health Sciences, University of Connecticut, Storrs, CT 06269, USA; 3 University of Connecticut Stem Cell Institute, University of Connecticut, Storrs, CT 06269, USA

**Keywords:** rheumatoid arthritis, collagen-induced arthritis, immune checkpoint molecule, TAPBPL, T cells, autoantibody

## Abstract

Rheumatoid arthritis (RA) is a chronic inflammatory autoimmune disease distinguished by synovial hyperplasia and a progressive destruction of joints. T cells are critical players in the pathogenesis of RA. We have previously identified a novel immune checkpoint molecule, TAPBPL, that inhibits T cell functions in vitro. As a model for human RA, we investigated the ability of the TAPBPL protein to ameliorate collagen type II (CII)-induced arthritis (CIA) in mice that were injected with recombinant TAPBPL or a control protein. The mice were analyzed for CIA development, immune cells, and their responses. We found that TAPBPL protein significantly decreased CIA incidence and reduced clinical and pathological arthritis scores, which were related to a lower number of activated CD4 T cells but a greater number of regulatory T cells (Tregs) in the spleen, and a reduction of Th1/Th17 inflammatory cytokines in the joints and serum. Importantly, TAPBPL protein inhibited CII-specific T cell growth and Th1 and Th17 cytokine expression and reduced the production of CII autoantibodies in the serum. Our results suggest that TAPBPL protein can ameliorate CIA in mice and has the potential to be used in the treatment of patients with RA.

## 1. Introduction

Rheumatoid arthritis (RA) is an often-encountered autoimmune disease distinguished by an immune reaction against synovial self-antigens, leading to inflammation of the synovial tissue, initiation of rheumatoid pannus, and erosion of nearby cartilage and bone, causing joint destruction [1,2,3,4]. Collagen-induced arthritis (CIA), induced in DBA/1 mice by type II collagen (CII), has been the most frequently employed animal model for human RA [5]. CII is a prominent protein in cartilage where RA is found. CIA has a number of pathologies in common with RA, such as synovial hyperplasia, mononuclear cell infiltration, and cartilage deterioration [5]. CIA animals continue to play an important role in pre-clinical studies, particularly for the identification and validation of new drug targets for human RA [5,6,7].

T cells are vital to the initiation and progression of this disease [8,9,10,11]. T cells are regulated by immune checkpoint molecules. Inhibitory checkpoint molecules are critical for maintaining peripheral tolerance to avoid autoimmune disease [12,13,14]. We previously reported the identification of a novel inhibitory immune checkpoint molecule TAPBPL (antigen-processing binding-protein-like molecule) [15]. TAPBPL shares significant sequence and structural similarities with existing checkpoint molecules [15]. TAPBPL protein is also expressed on the surface of T cells and antigen-representing cells [15]. The TAPBPL receptor can be found on CD4 and CD8 T cells, and other types of immune cells, and the expression levels are upregulated upon activation [15]. Recombinant TAPBPL protein significantly inhibits CD4 and CD8 T cell functions in vitro [15]. Administration of TAPBPL protein prevents and treats experimental autoimmune encephalomyelitis (EAE) in mice, an animal model for multiple sclerosis [15]. In this study, we investigated the ability of recombinant human TAPBPL protein to attenuate CIA in mice.

## 2. Results

### 2.1. TAPBPL Protein Reduces CIA Incidence and Symptoms

To determine the effect of TAPBPL on experimental arthritis, DBA/1 mice on day 0 were injected with CII emulated in complete Freund adjuvant (CFA) and on day 21 were given a booster of CII in incomplete Freund adjuvant (IFA). After a random division of the immunized mice, they were injected intraperitoneally (i.p.) with either a TAPBPL-constant region of mouse IgG2a (Ig) fusion protein (TAPBPL-Ig) or control Ig protein (25 or 50 µg) every third day for 30 days commencing from the day of the booster (day 21). We selected a dose of 50 µg for the control Ig group as no differences were observed between dosages of control Ig protein in preliminary studies.

**Figure 1:**
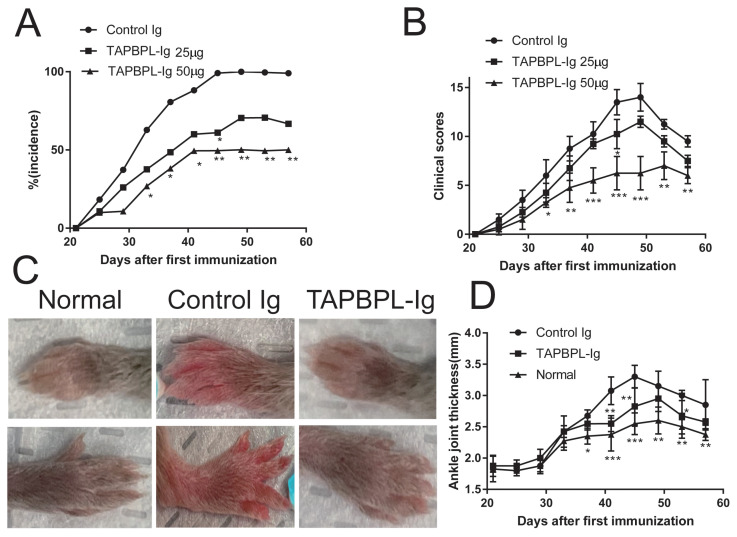
TAPBPL treatment reduces CIA incidence and clinical scores. CIA was generated in DBA/1 mice via injection with CII/CFA at day 0, followed by CII/IFA at day 21. TAPBPL-Ig protein (25 or 50 µg) or control Ig (50 µg) were given i.p. every third day for 30 days starting from day 21. (**A**) CIA occurrence and (**B**) clinical score are depicted. Pooled data are from 3 independent studies (15–18 mice per group). (**C**) Illustrative images of front and hind paws of normal mice with no treatment and control Ig- or TAPBPL-Ig-protein-treated CIA mice. (**D**) Statistical profile of ankle joint thickness in the hind paws. * *p* < 0.05, ** *p* < 0.01, *** *p* < 0.001 versus control Ig group.

TAPBPL-Ig significantly reduced the incidence of CIA, with about 70% of 25 µg TAPBPL-Ig-treated mice and 50% of 50 µg TAPBPL-Ig-treated mice developing CIA by day 58, whereas 100% of the control Ig-treated mice developed CIA (Figure 1A). TAPBPL-Ig also significantly decreased the clinical scores of arthritis in CIA mice with an average clinical score of 12 in the control Ig group, a score of 9 in the 25 µg TAPBPL-Ig-treated group, and a score of 6 in 50 µg TAPBPL-Ig-treated group on day 58 (Figure 1B). In addition, TAPBPL-Ig treatment delayed the disease peak time (Figure 1B). Figure 1C,D reveal that TAPBPL-Ig decreased the degree of joint swelling, as compared to control Ig treatment. An additional control was untreated normal mice (normal group).

### 2.2. TAPBPL Protein Ameliorates CIA Pathology

**Figure 2:**
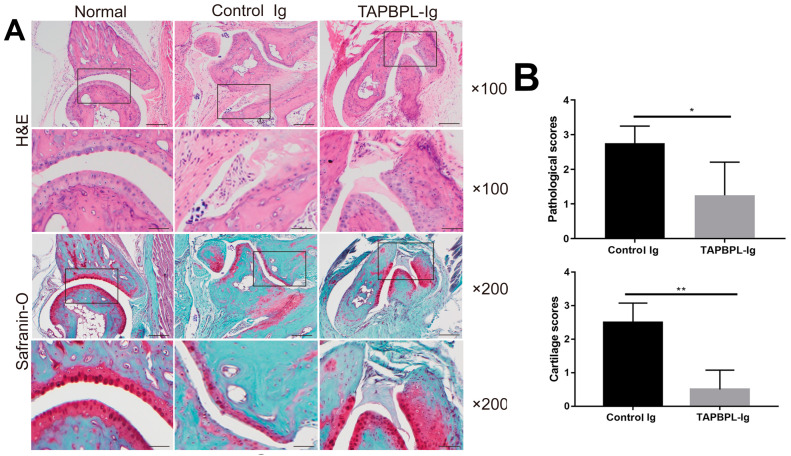
TAPBPL treatment reduces CIA pathology scores. Induction of CIA in DBA/1 mice and injection of control Ig or TAPBPL-Ig protein (50 µg) are as in Figure 1. The hind paw joints were harvested on day 58. (**A**) Hind paw examples of H&E (above) and Safranin-O (below) staining of control Ig- or TAPBPL-Ig-protein-treated CIA mice (50 µg groups). Hind paws from untreated normal mice were also used as a control. (**B**) Pathological scores were assessed via blind evaluation. Three independent experiments showed similar results (5–6 mice per group per experiment). * *p* < 0.05, ** *p* < 0.01 versus control Ig group.

Histological examination of arthritic paw joints stained with H&E from the control Ig-treated CIA mice demonstrated a severe infiltration of mononuclear cells, synovial hyperplasia, pannus formation, cartilage deterioration, and erosion of bone, all hallmarks of arthritis (Figure 2A). TAPBPL-Ig treatment, however, markedly reduced the levels of inflammation, infiltration, and cartilage erosion (Figure 2A). Safranin-O staining likewise revealed that the TAPBPL-Ig-treated CIA mice exhibited less damaged cartilage (Figure 2A). Consequently, TAPBPL-Ig treatment significantly decreased the pathological scores, as compared to those in the control Ig treatment (Figure 2B). Collectively, our results suggest that TAPBPL-Ig protein treatment ameliorates CIA in mice.

### 2.3. TAPBPL Protein Decreases the Production of Th1/Th17 Proinflammatory Cytokines in Joints and Serum

**Figure 3:**
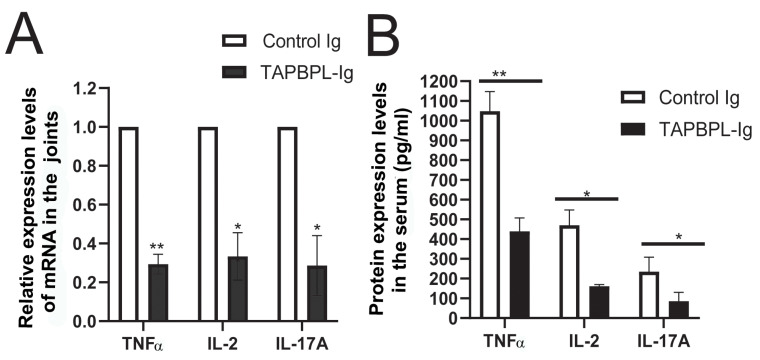
TAPBPL decreases the expression of Th1/Th17 proinflammatory cytokines in joints and serum. Post CIA induction, the DBA/1 mice received control Ig or TAPBPL-Ig protein (50 µg) as in Figure 2. The hind paw joints and serum were harvested on day 58. (**A**) RNA was isolated from the joint tissues and the expression levels of TNFα, IL-2, and IL-17A mRNA were analyzed via qRT-PCR. Number 1 defines levels of gene expression in control Ig-treated mice. (**B**) The expression levels of TNFα, IL-2, and IL-17A protein in the serum were analyzed via ELISA. Shown are mean ± SD from 1 of 3 independent studies with comparable results (5–6 mice/group/experiment). * *p* < 0.05, ** *p* < 0.01 versus control Ig group.

Research has shown that Th1/Th17 inflammatory cytokines perform a significant part regarding the initiation of arthritis [16,17,18]. We investigated whether TAPBPL-Ig impacted proinflammatory cytokine expression in the synovium of the CIA mice. TAPBPL-Ig significantly reduced the expression of TNFα, IL-2, and IL-17A mRNAs in the synovium (Figure 3A), as compared to control Ig-treated mice determined via real-time qualitative RT-PCR (qRT-PCR). We also analyzed the contents of these cytokine proteins in the serum via ELISA. TAPBPL-Ig also significantly reduced the amounts of TNFα, IL-2, and IL-17A protein in the serum of CIA mice (Figure 3B). The results suggest that TAPBPL treatment reduces the production of Th1/Th17 inflammatory cytokines in CIA mice.

### 2.4. TAPBPL Protein Reduces the Percentage and Activation of CD4 T Cells, but Augments the Percentage of Tregs in the Spleen

**Figure 4:**
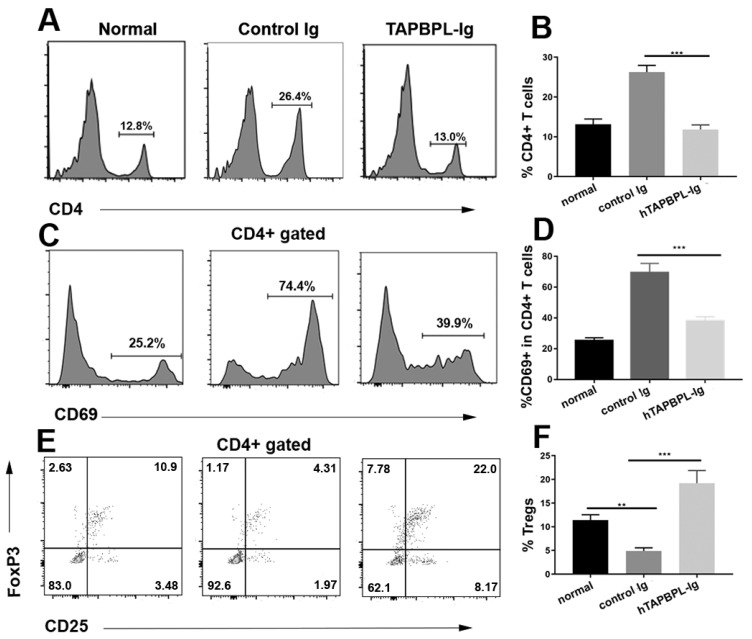
TAPBPL reduces the percentage and activation of CD4 T cells but raises the proportion of Tregs in the spleen. CIA-induced DBA/1 mice received control Ig or TAPBPL-Ig protein as in Figure 2. Spleens were collected on day 58. Untreated normal mice also served as controls. (**A**,**B**) The percentage of CD4^+^ T cells (**C**,**D**), CD69 expression by CD4^+^ T cells, and (**E**,**F**) the percentage of CD4^+^CD25^+^Foxp3^+^ Tregs in the spleen were analyzed. (**A**,**C**,**E**) Shown are flow cytometric images and (**B**,**D**,**F**) statistical analyses representing the collected data expressed as mean ± SD from 1 of 3 similar independent experiments (5–6 mice per group per experiment). ** *p* < 0.01, *** *p* < 0.001 versus control Ig group.

Since CIA is mainly initiated by CD4 T cells, mouse spleen CD4-positive T cells were analyzed. TAPBPL-Ig substantially lowered the percentage of CD4^+^ T cells (Figure 4A,B), additionally diminishing the activation marker CD69 through CD4-positive T cells (Figure 4C,D). It has been established that Tregs participate in immune tolerance induction, and that CD4^+^CD25^+^FoxP3^+^ cells are the best defined Tregs [19]; therefore, these cells in the spleen were also examined. TAPBPL-Ig significantly increased the percentage of Tregs (Figure 4E,F), consistent with our previous report in the EAE model [15]. Our data indicate that TAPBPL-Ig curtails the expansion and activation of CD4 T cells but increases the proportion of Tregs in CIA mice.

### 2.5. TAPBPL-Ig Protein Supresses CII-Specific T Cell Proliferation and Cytokine Production

**Figure 5:**
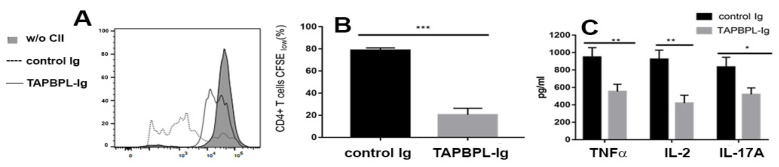
TAPBPL inhibits CII-specific T cell proliferation and cytokine production. CIA-induced DBA/1 mice were inoculated with control Ig or TAPBPL-Ig protein as in Figure 2. The spleens were collected on day 58. The splenocytes (normalized to 2 × 10^5^ T cells/well) were labelled with CFSE and stimulated with 25 µg/mL CII for 3 days in vitro. Splenocytes from untreated normal mice without (w/o) CII in vitro stimulation were also used as a control. (**A**,**B**) A CFSE dilution assay was used to measure CD4 T cell proliferation. (**A**) Representative flow cytometric images and (**B**) statistical analyses for proliferating CD4 T cells are shown. (**C**) Analyses for TNFα, IL-2, and IL-17A via ELISA were performed on supernatants of the cultured splenocytes stimulated with 25 µg/mL CII. Results are shown as mean ± SD from 1 of 3 similar independent experiments (5–6 mice per group per experiment). * *p* < 0.05, ** *p* < 0.01, *** *p* < 0.001 versus control Ig group.

To determine if TAPBPL reduces synovial autoantigen-specific T cell responses, splenocytes were harvested from the CIA mice and stimulated with CII in vitro for 72 h. T cell proliferative responses against CII were examined via carboxyfluorescein diacetate succinimidyl ester (CFSE) fluorescent dilution in CD4 T cells using flow cytometry. T cells in the spleen from the control Ig-treated mice responded to CII in vitro stimulation with an increase in proliferation (Figure 5A,B), while T cells in the spleen from TAPBPL-Ig-treated mice sustained a reduction in proliferation (Figure 5A,B).

We next examined the cytokine contents in the supernatants of the cultured splenocytes via an ELISA assay. A stimulative response to CII in vitro showed increased amounts of TNFα, IL-2, and IL-17A in the supernatants of the splenocytes from the control Ig-treated mice, whereas the production of these cytokines in the cells of TAPBPL-Ig-treated mice was largely reduced (Figure 5C). Collectively, our results indicate that TAPBPL-Ig decreases CII-specific T cell replication and Th1 and Th17 cytokine generation.

### 2.6. TAPBPL-Ig Protein Deminishes CII-Specific Autoantibody Production

**Figure 6:**
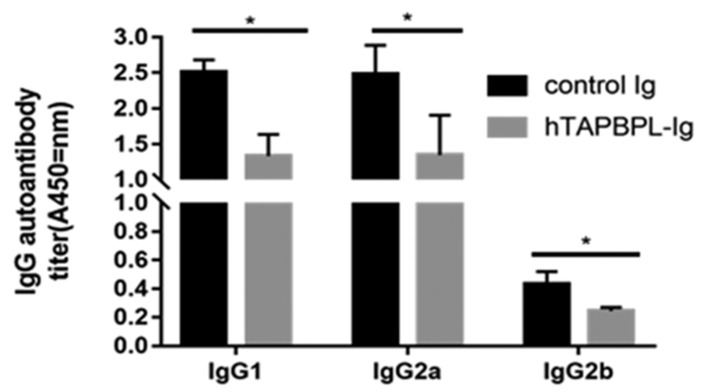
TAPBPL reduces CII-specific IgG1, IgG2a, and IgG2b antibody production in CIA mice. CIA-induced DBA/1 mice received control Ig or TAPBPL-Ig protein as in Figure 2. The blood was collected on day 58. Analysis of mouse serum for anti-CII IgG1, IgG2a, and IgG2b antibody production via ELISA. Results are given as mean ± SD from 1 of 3 similar independent experiments (5–6 mice/group/experiment). * *p* < 0.05 versus control Ig group.

Because CII autoantibodies are additionally involved in CIA pathogenesis [18,20], we analyzed the serum amounts of anti-CII antibodies. The serum levels of the anti-CII IgG1, IgG2a, and IgG2b antibodies of TAPBPL-Ig-treated mice were significantly reduced in comparison to those in the control Ig-treated mice (Figure 6). The results indicate that TAPBPL treatment also reduces the production of anti-CII antibodies in the CIA mice.

## 3. Discussion

We show here that the in vivo administration of TAPBPL-Ig significantly ameliorates CIA in mice. The beneficial effect is related to a decreased proportion and activation of CD4 T cells, but an increased proportion of Tregs and a reduced production of Th1/Th17 inflammatory cytokines. In addition, TAPBPL-Ig significantly decreased CII-specific T cell proliferation and cytokine output, and decreased CII-specific autoantibody levels.

We have previously shown that TAPBPL-Ig protein greatly attenuates the proliferation, activation, and cytokine production of both CD4 and CD8 T cells in vitro [15]. Here, we show that TAPBPL-Ig decreases the percentage CD4 T cells and inhibits CD4 T cell activation in CIA mice, consistent with our previous results [15]. Most importantly, TAPBPL-Ig curtails CII-specific CD4 T cell replication and Th1/Th17 cytokine output. We have also previously shown that although TAPBPL-Ig did not significantly decrease the percentage of CD8 T cells in EAE mice, it reduced the activation of CD8 T cells in this model [15]. However, in the current CIA model, we did not observe that TAPBPL-Ig significantly reduced the percentage and activation of CD8 T cells, which may be model-related. Our studies support the notion that CD4 T cells, particularly autoreactive CD4 T cells, are of major importance in CIA pathogenesis.

As opposed to downregulating effector CD4 T cell proliferation and activation, the addition of TAPBPL-Ig augmented the proportion of Tregs in CIA mice, which is consistent with our results in the EAE model [15]. It has been documented that native Tregs develop in the thymus, whereas induced Tregs generate in the periphery. Whether TAPBPL stimulates an increase in natural Tregs and/or induced Tregs has yet to be determined.

Locally produced Th1/Th17 inflammatory cytokines also participate in the pathogenesis of arthritis. Demonstrated here is that TAPBPL-Ig treatment decreases the production of inflammatory cytokines encompassing TNFα, IL-2, and IL-17A in both joints and serum. Other research has indicated that an interaction of immune cells with synovial fibroblasts (SFs) is also involved in RA pathogenesis [21,22]. TAPBPL might indirectly affect SFs by inhibiting the activation and proliferation of T cells. It is also possible that TAPBPL directly acts on SFs, which remains to be investigated.

CIA pathogenesis is also affected by B-cell-mediated autoimmune responses [20]. We have found that anti-CII IgG1, IgG2a, and IgG2b antibodies in serum are strikingly reduced in TAPBPL-Ig-treated CIA mice. The results indicate that TAPBPL also diminishes the generation of CII-specific autoantibodies from B cells. Because B cells also express the TAPBPL receptor [15], it could be that TAPBPL directly inhibits B cells to generate autoantibodies. Because CD4 T cells are important in B cell activation for antibody generation [23,24], it can also be speculated that the decreased generation of CII-specific autoantibodies in TAPBPL-Ig-treated CIA mice is caused by the inhibition of TAPBPL on CD4 T cells. Although male DBA/1 mice were used in the experiments for this paper, we have noted that TAPBPL-Ig protein significantly ameliorates CIA in female mice.

## 4. Materials and Methods

### 4.1. Mice

We obtained DBA/1 mice from Jackson Laboratory (Bar Harbor, ME, USA) and used male 4–8-week-old mice for the experiments. We housed the mice in groups of 2–5 mice/cage and maintained them under standard laboratory conditions: light from 7 A.M.–7 P.M., room temperature ~22 °C, humidity ~62%, low-fat rodent chow and tap water available ad libitum, and Beta Chip bedding changed twice a week. We treated the mice following the protocols approved by the Institutional Animal Care and Use Committee of the University of Connecticut.

### 4.2. Production of Recombinant Human TAPBPL-Ig Fusion Protein

We cloned the extracellular domain of the human TAPBPL gene into a pCMV6-AC-FC-S expression vector that contains the constant region of mouse IgG2a (ORIGENE, Rockville, MD, USA). We transfected the vector into HEK293F cells to produce the recombinant TAPBPL-Ig fusion protein. We purified the recombinant TAPBPL-Ig protein from the supernatant of cultured cells using Protein G Sepharose 4 Fast Flow, and verified the purified protein via SDS-PAGE, Coomassie staining, and Western blot as described [15]. We examined the endotoxin level in the purified protein and ascertained that it was less than 0.01 EU/mL of 1 µg of purified TAPBPL-Ig as determined via a Limulus Amebocyte Lysate assay [15].

### 4.3. CIA Induction

We injected DBA/1 mice intradermally at the tail base with 200 µg of bovine CII (Chondrex Inc., Woodinville, WA, USA) emulsified in CFA (Sigma Aldrich, St. Louis, MO, USA). Twenty-one days later, the mice were equally boosted with CII emulsified in IFA. After a random division of the immunized mice, we injected the mice i.p. with either TAPBPL-Ig or control Ig protein (25, or 50 µg) every 3 days for 30 days beginning from day 21. We recorded the onset of CIA and individual clinical scores. The swelling of four paws was scored from 0 to 4: grade 0, no sign of erythema and swelling; grade 1, erythema and slight swelling limited to tarsals or ankle joint; grade 2, erythema and slight swelling from ankle to tarsals; grade 3, erythema and moderate swelling from ankle to metatarsal joints; and grade 4, erythema and severe swelling involving the ankle, foot, and digits, or ankylosis of the limb [5]. Grading was performed for each paw, and the four scores were summed; the maximum score/mouse was 16. We euthanized the CIA mouse if (1) weight loss was ≥25%, (2) they were unable to eat or drink, (3) there was evidence of joint swelling >50% of baseline, (4) there was evidence of self-trauma to the joint, (5) excessive skin breakdown occurred in arthritic joints, or (6) the general health of the mouse was at a level requiring euthanasia for humane reasons.

### 4.4. Histological Assessment of Arthritis

We fixed the rear paws in 10% neutral buffered formalin and decalcified them in 10% EDTA. We sectioned the tissues and stained them with H&E. Blind evaluation of all slides was carried out. The extent of synovitis, pannus formation, and bone/cartilage destruction was assigned using the following grading scale [25]: grade 0, no indication of inflammation; 1, mild inflammation with synovial lining hyperplasia and no cartilage deterioration; 2–4, increasing inflammatory cell infiltration and cartilage/bone destruction. Safranin-O staining on tissue sections was used to determine the cartilage disintegration with a 0–4 score system as described [26]: 0 = no deterioration; 1 = minimal erosion; 2 = slight to moderate erosion in a limited area; 3 = more extensive erosion; 4 = general disintegration.

### 4.5. CII-Specific T Cell Proliferation and Cytokine Production

We labeled erythrocyte-depleted splenocytes with CFSE (ThermoFisher Scientific, Waltham, MA, USA) and cultured the cells with denatured (60 °C, 30 min) bovine CII for 72 h. We analyzed T cell proliferation by determining CFSE fluorescent intensity in CD4^+^ or CD8^+^ T cells with flow cytometry. We also collected the supernatants of the cultured cells after 72 h and measured cytokine presence via ELISA (Biolegend, San Diego, CA, USA).

### 4.6. Flow Cytometry Analysis

The single-cell suspension of organs was stained with the fluorochrome-conjugated antibodies as described [27,28,29,30]. As for intracellular staining, cell permeabilization was accomplished using a BD Cytofix/Cytoperm solution for 20 min at 4 °C. We then stained the cells with fluorochrome-conjugated antibodies including CD4, CD8, CD25, Foxp3, and CD69 (BioLegend or BD Biosciences, San Diego, CA, USA). We used an LSRFortessa X-20 Cell Analyzer (BD Biosciences) for sample analysis, and FlowJo software v10.6 (Ashland, OR, USA) for data analysis.

### 4.7. qRT-PCR

We synthesized cDNA from total RNA that was isolated from tissues or cells, and performed RT-PCR using equal quantities of cDNA as described [31]. We carried out qRT-PCRs using the Power SYBR green mastermix (Applied Biosystems, Waltham, MA, USA) along with the 7500 real-time PCR system (Applied Biosystems) [13].

### 4.8. Measurement of Serum Anti-CII Antibody and Cytokine Levels

We coated bovine CII (1 µg/mL) onto microtiter plates overnight at 4 °C. After blocking with 1% BSA in PBS, we added serum samples to the plates and incubated them at room temperature for 1 h. After washing, we added biotin-conjugated anti-mouse IgG1, IgG2a or IgG2b antibody (Biolegend), and then HRP-labelled avidin (Biolegend). We used a TMB Substrate Set (Biolegend) to detect antibody binding according to the manufacturer’s instructions. An ELISA Kit (Biolegend) was used to measure TNFα, IL-2, and IL-17A protein concentrations.

### 4.9. Statistical Analysis

We used a two-sided Student’s t test to determine *p*-values. For comparing means of multiple groups, we used one-way ANOVA with Dunnett test to determine significance. A confidence level greater than 95% (*p* < 0.05) was deemed significant.

## 5. Conclusions

We have verified that the in vivo administration of human TAPBPL protein significantly ameliorates CIA in mice due to a decreased expansion and activation of effector CD4 T cells, especially CII-specific CD4 autoreactive T cells, but also an increased production of Tregs. Therefore, TAPBPL protein stands out as a potential treatment for patients with RA.

## Data Availability

The data generated during and/or analyzed during the current study are available from the corresponding author on reasonable request.

## Abbreviations

TAPBPL: antigen-processing binding protein-like molecule; RA: rheumatoid arthritis; CII: collagen type II; CIA: collagen type II-induced arthritis; CFSE: carboxyfluorescein diacetate succinimidyl ester; EAE: experimental autoimmune encephalomyelitis; qRT-PCR: real-time qualitative RT-PCR; Tregs: regulatory T cells; CFA: complete Freund adjuvant; IFA: incomplete Freund adjuvant.
